# Identified micro-organisms in hospitalized community-acquired pneumonia patients living near goat and poultry farms

**DOI:** 10.1186/s41479-021-00091-w

**Published:** 2021-11-29

**Authors:** Inge Roof, Arianne B. van Gageldonk-Lafeber, Tizza P. Zomer, Yolande M. Vermeeren, Peter C. Wever, Wim van der Hoek

**Affiliations:** 1 Centre for Infectious Disease Control, National Institute for Public Health and the Environment (RIVM), Bilthoven, The Netherlands; 2 Department of Medical Microbiology and Infection Prevention, Gelre Hospital, Apeldoorn, the Netherlands; 3 Department of Internal Medicine, Gelre Hospital, Apeldoorn, the Netherlands; 4 Department of Medical Microbiology and Infection Control, Jeroen Bosch Hospital, ’s-Hertogenbosch, the Netherlands

**Keywords:** Community-acquired pneumonia, Etiology, Goat farms, Poultry farms, Livestock

## Abstract

**Background:**

In the Netherlands, an increased risk of community-acquired pneumonia (CAP) has been reported for adults living near goat and poultry farms. Previous results of respiratory microbiome studies in hospitalized CAP patients near poultry farms suggested a higher relative abundance of *Streptococcus pneumoniae.* This retrospective study, using routine laboratory data from hospitalized CAP patients, aims to explore possible aetiologic micro-organisms of CAP in relation to livestock exposure.

**Methods:**

Patient characteristics and PCR and urinary antigen test results were retrieved retrospectively from electronic medical records of CAP patients admitted to the Jeroen Bosch Hospital or Gelre Hospital in the Netherlands during 2016–2017. Distances between the patients’ home address and the nearest poultry and goat farm were calculated. Differences in laboratory test results between CAP patients with and without goat or poultry farms within 2 km of their home address were analyzed using Fisher’s exact test.

**Results:**

In total, 2230 CAP episodes with diagnostic results were included. In only 25% of the CAP episodes, a micro-organism was detected. A positive urinary antigen test for *S. pneumoniae* was found more often in patients living within two kilometers of goat (15.2% vs. 11.3%) and poultry farms (14.4% vs. 11.3%), however these differences were not statistically significant (*p* = 0.1047 and *p* = 0.1376).

**Conclusion:**

Our retrospective analysis did not show statistically significant differences in the identified micro-organisms in hospitalized CAP patients related to livestock farming. The study was hampered by limited statistical power and limited laboratory results. Therefore, the potential increased CAP risk around goat and poultry farms will be further explored in a prospective study among CAP patients in primary care.

## Introduction

In the Netherlands, large numbers of livestock animals are kept in close proximity to human residential areas. This gave rise to concerns among local residents and public health professionals. Previous large epidemiological studies in the Netherlands showed an increased risk of community-acquired pneumonia (CAP) among adults living within a 1–2 km range of goat and poultry farms. [1–5] Based on data from electronic medical records, the association with general practitioner (GP) diagnosed CAP has been repeatedly present from 2009 to 2016 for goat farms. Although the association with poultry farms has also been found in the United States [6], in more recent studies in the Netherlands the associations with poultry farms were not significant or positive in only a part of the analyses. [2, 7] During 2014–2016, an estimated 6.0 to 7.8% of CAP cases in a study population in the south of the Netherlands were attributable to living in the vicinity of goat farms. [5] The association between CAP and goat farms led to a temporary stop on issuing building permits for new and existing goat farms in most Dutch provinces. [8]

CAP is mostly a clinical diagnosis with only limited information on the aetiologic micro-organisms. It has been proposed that exposure to particulate matter (PM) and its components (e.g. micro-organisms and endotoxins) may increase the susceptibility to CAP. [9–11] Previous results suggested a shift in the respiratory microbiome composition in hospitalized pneumonia patients near poultry farms, with a higher relative abundance of *Streptococcus pneumoniae*. [3] While most CAP patients in the Netherlands are managed by the GP, yearly, around 40,000–50,000 pneumonia patients in the Netherlands require hospitalization. Even in hospitalized CAP patients, the causative organism is not specified in 85% of cases when routine diagnostics are applied [12]. Despite this limitation, in preparation for a large prospective aetiological study, we still decided to analyze the laboratory management systems of two hospitals in livestock dense areas for relevant microbiological test data, especially for *S. pneumoniae*, to explore the identified micro-organisms in pneumonia patients in relation to livestock exposure.

## Methods

### Study design and data collection

We used laboratory data from CAP patients admitted to Jeroen Bosch Hospital (JBH) or Gelre Hospital (Gelre) in the Netherlands during the period Jan 1, 2016 – Dec 31, 2017. These hospitals were chosen based on their location within livestock dense areas. Within the catchment area of JBH live 360.000 people. The catchment area of Gelre includes approximately 280.000 inhabitants. Patients were selected based on financial reimbursement codes (DBC, Dutch acronym: Diagnose Behandel Code) specific for CAP. Reimbursement codes for CAP in children (age below 18 years) were excluded. No ethical approval was needed as patient records were used retrospectively. The variables age, gender, postal code and date of sample collection were retrieved from the electronic patient record and combined with laboratory test results. For both hospitals, results were available from PCR on influenza virus A and B, and from urinary antigen tests for *Legionella pneumophila* serogroup 1 and *S. pneumoniae.* Additionally, data from Gelre hospital included test results from a PCR panel on human metapneumovirus, adenovirus, *Chlamydia pneumoniae*, rhinovirus, coronavirus, *Chlamydia psittaci* and *Mycoplasma pneumoniae*. Test results from the same patient with an interval of more than 1 month were considered as a new CAP episode. For each episode the variable ‘diagnosis’ was added and was considered positive when at least one micro-organism, irrespective of the type, was reported in any of the laboratory test results.

### Livestock farm exposure

We obtained the goat and poultry farm locations from the Netherlands Enterprise Agency, which collects livestock farm data on behalf of the Ministry of Agriculture, Nature and Food Quality. The location and characteristics of the goat and poultry farms were available for April 2016 and April 2017. The year of livestock farming data used, corresponded with the year of hospital admission. Only livestock farms with a minimum number of 50 goats and 250 poultry birds were included. The postal codes of patients’ residential addresses were geocoded and distances between these coordinates and the nearest poultry and goat farm were calculated using ArcGis. A binary variable, indicating the presence of a farm of a specific type within two kilometers of the home address, was created. The cut-off of two kilometer was selected based on the results of previous studies. [2, 5]

### Statistical analysis

Descriptive statistics were performed with categorical variables presented as percentages and continuous variables as median with interquartile range. Differences in categorical and continuous variables were calculated using Fisher’s exact test and the Wilcoxon rank-sum test, respectively. All data were analyzed using SAS software version 9.4 (SAS Institute Inc., Cary, NC, USA). A *p*-value of 0.05 was considered statistically significant.

## Results

### Characteristics CAP episodes

For the years 2016–2017, a total of 2230 CAP episodes (*n* = 1275 JBH, *n* = 955 Gelre) with diagnostic test results for PCR and/or urinary antigen tests were included, representing 1928 unique patients. Of the total number of CAP episodes, 12.4% (JBH 19.1%, Gelre 3.6%) and 16.6% (JBH 18.2%, Gelre 14.5%) occurred in patients living within a two kilometer range of goat farms or poultry farms, respectively (Table 1). A similar age distribution was observed between the CAP patients with and without livestock presence. The group living close to poultry farms consisted of a higher percentage of males (*p* = 0.0113). Diagnostic tests were able to detect a micro-organism in around a quarter of the CAP episodes in each livestock exposure group. No associations were found between season of admission and goat or poultry farm presence (Table 1).

**Table 1:** Characteristics of hospitalized pneumonia patients, grouped by livestock farm exposure

	Goat farm ≤2 km N (%)	Goat farm > 2 km N (%)	P-value	Poultry farm ≤2 km N (%)	Poultry farm > 2 km N (%)	*P*-value
N	277 (12.4)	1953 (87.6)		370 (16.6)	1860 (83.4)	
Median age, years [IQR]	73 [18]	72 [19]	0.1740	72 [20]	72 [19]	0.9810
Male gender	162 (58.5)	1121 (57.4)	0.7458	235 (65.5)	1048 (56.3)	**0.0113**
Positive diagnosis	66 (23.8)	493 (25.2)	0.6569	100 (27.0)	459 (24.7)	0.3579
Season of admission						
Apr-Sep	108 (39.0)	708 (36.3)	0.3866	135 (36.5)	681 (36.6)	1.0000
Oct-Mar	169 (61.0)	1245 (63.7)		235 (63.5)	1179 (63.4)	

### Micro-organisms identified

Within the laboratory data, PCR test results were available for influenza virus A and B (*n* = 1109) and urinary antigen test results for *L. pneumophila* serogroup 1 (*n* = 1837) and *S. pneumoniae* (*n* = 1799). Comparing patients exposed and non-exposed to goat or poultry farms within two kilometers of their home address, showed no statistically significant differences with regard to test results for influenza virus A and B, and *L. pneumophila* serogroup 1 (Table 2). Higher percentages of positive urinary antigen tests for *S. pneumoniae* were found for patients living close to goat (15.2% vs. 11.3%) and poultry farms (14.4% vs. 11.3%), however, these differences were not statistically significant (Table 2, *p* = 0.1047 and *p* = 0.1376). When analyzing CAP episodes from JBH separately, a positive urinary antigen test for *S. pneumoniae* was significantly associated with poultry farm exposure (*p* = 0.0318, data not shown). Analysis of the PCR panel for CAP episodes from the Gelre Hospital showed no differences in any of the included micro-organisms (data not shown).

**Table 2:** Laboratory results of PCR and urinary antigen tests of pneumonia patients, grouped by livestock farm exposure

		Goat farm ≤2 km N (%)	Goat farm > 2 km N (%)	P-value	Poultry farm ≤2 km N (%)	Poultry farm > 2 km N (%)	*P*-value
Influenza A PCR	Pos Neg	17 (17.0) 83 (83.0)	152 (15.1) 857 (84.9)	0.5628	32 (18.0) 146 (82.0)	137 (14.7) 794 (85.3)	0.2574
Influenza B PCR	Pos Neg	6 (5.9) 95 (94.1)	27 (2.7) 981 (97.3)	0.1125	5 (2.8) 173 (97.2)	28 (3.0) 903 (97.0)	1.0000
Legionella urinary antigen test^*^	Pos Neg	7 (2.9) 239 (97.1)	29 (1.8) 1562 (98.2)	0.3175	8 (2.7) 288 (97.3)	28 (1.8) 1513 (98.2)	0.3561
Pneumococcal urinary antigen test^#^	Pos Neg	36 (15.2) 201 (84.8)	177 (11.3) 1385 (88.7)	0.1047	42 (14.4) 249 (85.6)	171 (11.3) 1337 (88.7)	0.1376

^*^Legionella pneumophila serogroup 1

^#^*Streptococcus pneumoniae*

## Discussion

This retrospective analysis of laboratory tests in hospitalized CAP patients did not show significant differences in the identified micro-organisms between patients with and without goat and poultry farms in the vicinity of their home address.

Based on previous research, in which a shift in the respiratory microbiome with higher abundance of *S. pneumoniae* in hospitalized CAP patients close to poultry farms was observed [3], we hypothesized to find a higher percentage of positive tests for *S. pneumoniae* in patients exposed to livestock. However, in our study a positive urinary antigen test for *S. pneumoniae* was not significantly associated with poultry or goat farm presence around the patients’ home address. An explanation might be that we used results from urinary antigen tests, a relative insensitive test compared to the 16S-rRNA based sequencing to determine *S. pneumoniae* presence used in the study by Smit et al. [3] Moreover, Smit et al. used a one kilometer distance as cut-off point for poultry farm presence instead of the two kilometers distance we defined. The number of people living within one kilometer of goat or poultry farms in our study was too low for proper statistical analysis. When analyzing the urinary antigen tests for CAP episodes from JBH only, we did find a statistically higher percentage of positive urinary antigen tests for *S. pneumoniae* in patients living close to poultry farms (15.6%) compared to patients living more than two kilometers from poultry farms (10.1%). Compared to Gelre hospital, urinary antigen tests were more often performed at JBH and a higher percentage of patients from JBH live within two kilometers of a livestock farm.

Prior studies regarding CAP and proximity to livestock farming used electronic medical records from GPs, reflecting mostly outpatient CAP. Our present study was among hospitalized CAP patients, i.e. a more severe patient population. The identified micro-organisms in these hospitalized patients might not entirely reflect the etiology of CAP patients visiting their GP. No patterns with goat or poultry farm exposure were detected for the micro-organisms included in a respiratory PCR panel (e.g. human metapneumovirus, adenovirus, rhinovirus, coronavirus, *C. pneumoniae, C. psittaci* and *M. pneumoniae*). Because of differences in diagnostic testing strategies, results from this PCR panel were only available for CAP episodes from Gelre Hospital. Unfortunately, the low prevalence of goat farms around the home addresses of these patients (3.6%) hampered the analyses of these micro-organisms due to limited statistical power.

In around 75% of the CAP episodes included in our study, no pathogens were detected. The failure to detect a pathogen has often been described in scientific literature, with estimates ranging from 20 to 45% of patients. [13–17] The high percentage of diagnostic failure reported in this study, might be related to the fact that our data was limited to routine diagnostics performed in these patients. A comprehensive and broad diagnostic package can increase diagnostic yield. However, we did not include test results from serology and bacterial culture, because these results often require an extra interpretation step.

During the large Q-fever epidemic in the Netherlands, from 2007 to 2010, *Coxiella burnetii*, was clearly an important zoonotic cause of CAP among people living close to Q-fever-affected goat farms. An observational study on CAP etiology in the Netherlands, that coincided with this Q-fever outbreak, showed that *C. burnetii* was the second most common pathogen in that study population. [16] However, in more recent years, after the epidemic, it was unlikely that Q-fever still played a role. *C. burnetii* seropositivity among CAP patients was independent from goat farm exposure and the increased CAP incidence was also found around Q-fever negative farms. [2, 4] In our study, very little to no Q-fever diagnostics had been requested in the CAP episodes included from the JBH, the hospital which is located within the former Q-fever epidemic area.

Our study was limited by multiple factors. First, the analysis was hampered by the small number of identified micro-organisms and the low prevalence of livestock exposure around some of the home addresses of patients. Therefore we only analyzed the laboratory results in relation to livestock farm exposure by crude univariate analysis. The small numbers and low prevalence also did not allow for further classification of the livestock exposure variable. We chose to handle a 2 km distance cut-off based on the results from previous research, but further classification using buffers or regression frameworks, as used in previous studies, was not appropriate. Second, as we did not have additional patient characteristics such as comorbidities, lifestyle factors and medication use, we could not address potential confounding by certain risk factors in our analyses. The age of the CAP patients was similarly distributed between the groups with and without livestock farm proximity. Finally, potential selection bias is also of concern because not every patient was tested for each pathogen. We retrospectively used laboratory data initially performed for diagnostic purposes, meaning that the data is dependent on the prevailing testing strategies in both hospitals. It might be that physicians only test for certain pathogens in the more severe patient group. Still the overall pathogen detection rate of around 25%, as presented in Table 1, was comparable between the CAP patients with and without goat and poultry farm exposure.

This explorative study did not provide information about possible causes of the increased CAP risk around goat and poultry farms. The previous associations are based on diagnostic data from GPs. In the Netherlands, around 80% of all CAP patients are managed by the GP. The causative pathogen, however, remains unknown in the majority of CAP patients as microbiological testing is not included in the pneumonia guidelines of the Dutch College of General Practitioners. [18, 19] To gather insight in CAP etiology related to livestock farming in a primary care setting, we started a prospective study in CAP patients visiting their GP including extensive microbiological diagnostics by multiplex PCR and respiratory microbiome analyses.

## Data Availability

The datasets used and/or analyzed during the current study are available from the corresponding author on reasonable request.

## Abbreviations

CAP: Community-acquired pneumonia
GP: General practitioner
PM: Particulate matter
JBH: Jeroen Bosch Hospital
Gelre: Gelre Hospital
